# Drought, herbivory and combined stress caused treatment-specific changes in phytohormones, but species-specific changes in secondary metabolite profiles in thyme

**DOI:** 10.3389/fpls.2025.1711338

**Published:** 2025-12-01

**Authors:** Maryam Alipour, Laure Martinelli, Karin Groten, Axel Mithöfer

**Affiliations:** 1 Research Group Plant Defense Physiology, Max Planck Institute for Chemical Ecology, Jena, Germany; 2 Department of Horticulture, College of Agriculture, Isfahan University of Technology, Isfahan, Iran

**Keywords:** thyme, herbivory, drought, secondary metabolites, phytohormones, gene expression

## Abstract

Thyme species are important medicinal herbs predominantly cultivated in arid regions. They increasingly experience the adverse effects of climate change, particularly drought. Although abiotic stress effects on thyme have been examined, limited information exists on insect herbivory or combined stress response. This study investigated the effects of drought stress, herbivory and their combination on secondary metabolism, phytohormone regulation and insect performance in three thyme species: *Thymus serpyllum* (drought-sensitive), *T. kotschyanus* (drought-tolerant), and *T. vulgaris*. Plants were exposed for three weeks to four treatments: control, drought (40% field capacity), *Spodoptera littoralis* larvae herbivory, and combined drought × herbivory. Herbivore performance was documented. Levels of volatile terpenes, phenolic compounds, and phytohormones were analyzed using GC-MS and LC-MS/MS. Gene expression of terpenoid biosynthesis enzymes (*TPS2, CYP71D178, CYP71D181*) was quantified via RT-qPCR. Results revealed species-specific responses in secondary metabolism and measured gene expression, with herbivory exerting stronger effects than drought. Combined stress triggered the strongest responses in *T. vulgaris* and *T. serpyllum*, whereas *T. kotschyanus* tended to respond more to individual stressors. In contrast to species-specific metabolic responses, phytohormones exhibited treatment-specific patterns, with herbivory inducing the most pronounced changes across all measured phytohormones. Hormonal and metabolic adaptations were associated with reduced larval performance on stressed plants. Overall, the results show that the thyme species studied here respond to drought and herbivory through species-specific modulations of secondary metabolism. The integration of transcriptome, metabolome and phytohormone data provides new insights into the dynamic responses of Mediterranean aromatic plants to abiotic, biotic and combined stress factors, with implications for plant protection and resilience under climate change scenarios.

## 1 Introduction

Plants constantly encounter various abiotic and biotic stresses, among which drought is one of the most limiting factors affecting productivity. Global climate change is projected to intensify droughts and heat waves, severely affecting both natural ecosystems and agricultural productivity (Sato et al., 2024). In response to water limitation, plants undergo profound physiological, biochemical, and molecular changes, including reduced photosynthetic activity, increased reactive oxygen species (ROS) production, activation of antioxidant systems, and reprogramming of primary and secondary metabolism (Lin et al., 2023; Sun and Fernie, 2024; Cao et al., 2024). Phytohormones, such as abscisic acid and ethylene play pivotal roles in drought-induced signaling and adaptation (Sahu and Giri, 2025). The severity and duration of stress, as well as the species and individual genotypes, influence plant responses (Tuberosa, 2012). Drought rarely acts in isolation; drought-induced physiological and metabolic alterations often interact with other stressors, such as insect herbivory. Herbivory activates jasmonic acid (JA)-dependent signaling that regulates defense gene expression and metabolite synthesis (Erb and Reymond, 2019; Lin et al., 2023; Sahu and Giri, 2025). Outbreaks of herbivorous insects often coincide with drought stress (Lin et al., 2023). Various theories have been proposed regarding how these two stress factors may interact, but the outcomes can vary. The interaction outcome may result in additive, neutral, or dominant effects, depending on the stress combination (Sahu and Giri, 2025). Ultimately, the outcome depends on specific plant–herbivore interactions (Lin et al., 2023). However, the interplay between drought, herbivory, and metabolite production remains poorly understood in most plant species.

The cotton leafworm, *Spodoptera littoralis*, is a polyphagous lepidopteran pest that can severely damage a wide variety of host plants, including aromatic herbs. *S. littoralis* feeding rapidly activates JA-signaling and associated transcription factors, which thereby orchestrating the biosynthesis of defensive metabolites (Leitner et al., 2005).

The genus *Thymus* (Lamiaceae) includes ~350 species native to Northern Africa and Eurasia. Its essential oils, rich in monoterpenes such as thymol and carvacrol, hold significant medicinal and industrial importance and serve as chemical defenses against herbivores and pathogens (Mahdavi et al., 2020). Thymol and carvacrol are toxic to several insects, including *S. littoralis*, and *Plutella xylostella* (Pavela, 2014; Kumrungsee et al., 2014; Agliassa and Maffei, 2018). These compounds are synthesized through the terpenoid biosynthesis pathway, involving key genes such as geranyl diphosphate synthase (*GPPS*), *γ*-terpinene synthase (*γ-TPS*), cytochrome P450 monooxygenases, and terpene synthases (Crocoll, 2011). The expression of these genes is regulated by hormonal signals, particularly JA, salicylic acid (SA), and ABA, whose regulation is influenced by environmental stressors (Crocoll, 2011; Tholl, 2015). In addition to terpenes, phenolic compounds synthesized via the phenylpropanoid pathway play a key role in antioxidant defense and herbivore deterrence in thyme and other species (Dixon and Paiva, 1995).

Different thyme species are known to differ in drought tolerance, and this variation may be associated with their capacity to accumulate particular primary and secondary metabolites (Ashrafi et al., 2022). Previously, it was shown that species vary in their levels of terpene production under both drought stress and non-stress conditions (Alipour et al., 2025). Some species with higher drought tolerance accumulate metabolites than those that are sensitive to drought (Mansinhos et al., 2022). In contrast, *T. kotschyanus* accumulated more terpenes due to drought stress (Alipour et al., 2025), as did the drought tolerant variety *T. vulgaris* Varico3 (Mahdavi et al., 2020). Despite recent advancements in our understanding of drought stress responses in thyme species, our knowledge of thyme’s response to insect herbivory remains limited. An early study demonstrated that the growth of *T. vulgaris* is impaired after aphid feeding (Linhart et al., 2005), and other herbivorous species (two mollusks, two insects, and two mammals) respond specifically to the chemical profiles of different chemotypes of *T. vulgaris* (Linhart and Thompson, 1999).

However, to the best of our knowledge, no previous studies have investigated changes in chemical profiles and phytohormone level. Moreover, the interaction between drought and herbivory in *Thymus* species remains largely unexplored. Therefore, this study employed LC/GC-MS and RT-qPCR analyses to address the following research question: What are the changes in terpenoid and phenolic profiles, as well as in phytohormone levels in response to insect herbivory? Does this response change with the simultaneous application of drought stress? Does the drought tolerance of a particular species affect its response? To answer these questions, we compared the drought-tolerant species *T. kotschyanus* and the drought-sensitive species *T. serpyllum* with *T. vulgaris* as a reference.

## 2 Materials and methods

### 2.1 Plants and insects

Seeds of three different thyme species, *T. serpyllum*, *T. kotschyanus*, and *T. vulgaris*, were provided by the Research Institute of Forests and Rangeland in Tehran, Iran. The seeds germinated in a seedling plastic tray with a 1:1 blend of peat moss and coco peat. The trays were placed in a growth chamber (York) equipped with LED NS1 lights (Valoya, Norway). After two months, the seedlings were transplanted into plastic pots (10 cm in diameter and 8 cm in height, with five seedlings per pot) filled with the five components: sieved soil, sand-clay granule mix, perlite, vermiculite, and lecaton in the following ratio 3:2:1:0.5:0.5. Plants were kept at 22°C, and growth chamber settings included a 16-h photoperiod, 35-50% relative humidity, and 180 mmol m^-2^, mimicking day-light conditions.

Eggs of the generalist herbivore *Spodoptera littoralis* Boisd. (Lepidoptera, Noctuidae) were obtained from Syngenta Crop Protection AG (Switzerland). Larvae were hatched and reared on an artificial diet with a 10 h light/14 h dark photoperiod at 23 to 25°C (Bergomaz and Boppré, 1986).

### 2.2 Treatment

All plants were irrigated daily for two months. After full establishment at the vegetative growth stage, the plants were subjected to drought stress for a period of three weeks at 100% and 40% (C = control and D = drought stress, respectively) of field capacity (FC). To calculate the amount of water required per pot for each irrigation regime, the soil FC was measured at the beginning of the experiment using the weighing method (Pourmeidani et al., 2017). First, the pots were filled with soil and thoroughly irrigated to achieve a saturated soil. To prevent evaporation, the pot was covered with plastic, and after 24 h, the pots were weighed every two hours. The pots were weighed when their weights were fixed. The percentage of water in the soil under FC conditions was determined using the following equation:


Soil water content(%)=[(soil fresh weight−soil dry weight)/soil dry weight]×100

After subtracting the weight of the pot and dry soil, the amount of water held under FC conditions was calculated, and different irrigation treatments (100% and 40% FC) were calculated accordingly. To this end, the pots were weighed daily using the prescribed and calculated weight for each treatment, and the appropriate amount of water was added to each pot.

### 2.3 Herbivore performance

To analyze *S. littoralis* performance on each thyme species, eight potted plants at the same phenological stage were selected. Six first-instar larvae were carefully transferred to each plant. Each pot was enclosed within a perforated transparent plastic bag to prevent larvae from escaping while ensuring adequate ventilation. The pots were maintained in a growth chamber under the same controlled conditions as described above. The survival and biomass of the larvae were recorded on various specified days after the infestation of well-watered plants. The total number of living larvae on each plant was counted, and all surviving larvae from each pot were weighed together using a precision electronic balance with an accuracy of 0.1 mg. Survival was calculated as the proportion of live larvae relative to the initial number (n = 6 per pot).

In an independent set of experiments, 3^rd^ instar *S. littoralis* larvae were placed on both drought-stressed and well-watered plants of all three species and allowed to feed for 24 h before the plant leaves were harvested into liquid nitrogen and subjected to further analyses. Thus, plants were subjected to one of four treatments: (1) control: without drought or herbivory stress (C), (2) drought-stress only (D), (3) herbivore infestation only (H), and (4) combined drought-stress and herbivore infestation (HD).

### 2.4 RNA extraction, cDNA synthesis and qRT-PCR analysis

Gene expression analyses were performed as previously described (Alipour et al., 2025). Briefly, total RNA from thyme leaves was extracted from 100 mg of powdered leaves using the InviTrapR Spin Plant RNA Mini Kit (Invitek Molecular GmbH) according to the manufacturer’s instructions. On-column DNA digestion with DNaseI (TURBO DNA-freeTM kit) was performed. The concentration, purity, and quality of RNA were measured with a spectrophotometer (NanoDrop, 2000c; Thermo Scientific). The RevertAid First Strand cDNA Synthesis Kit (Thermo Scientific) was used to synthesize cDNA from 900 ng μL RNA^-1^. Oligo (dT) 18 primers were used according to the manufacturer’s recommendations.

We used the normalized expression method (fold change) to analyze the relative transcript values of monoterpene synthase genes, including *γ*-terpinene synthase (*TPS2*), thymol synthase (*CYP71D178* and *CYP71D181*), and carvacrol synthase (*CYP71D181*). Each sample had three biological and two technical replicates. Specific primers for qRT-PCR were constructed based on previously obtained conserved sequencing data of thyme species, and elongation factor (*EF1*) was used as a reference gene for normalization. Real-time PCR was performed using an iCycler (Bio-Rad) with SYBR Green II (Agilent Technologies). The qRT-PCR settings were set as follows: an initial denaturation phase at 95°C for three min, followed by 40 cycles of denaturation at 95°C for 30 s, annealing at 57°C for 35 s, and a final elongation step at 72°C for 30 s. The primers used in the qRT-PCR procedures were gene-specific and have been successfully employed previously (Alipour et al., 2025). These are shown in **Supplementary Table S1** (Crocoll, 2011; Tohidi et al., 2020; Ashrafi et al., 2022). Normalized raw Ct values were used to compare the results of control and treatment sample results using the 2^−ΔΔCt^ (fold change) method, as previously described (Livak and Schmittgen, 2001).

### 2.5 Terpenoid analysis by GC-MS

Samples of young leaves from each species were collected independently, frozen in liquid nitrogen, and ground into a fine powder using a mortar and pestle. Then, 50 mg of the plant powder was extracted with 400 µL of hexane (containing 10 µg mL^-1^ of the internal standard nonyl acetate) in a 1.5 mL glass vial at room temperature for one hour. Fifty µl of the hexane phase were transferred to a new glass vial for GC-MS analysis (Krause et al., 2021). The extract underwent gas chromatography using an Agilent 6890 series gas chromatograph (Agilent Technologies), with a 1 µL splitless injection and a flow rate of 2 mL min^-1^ using helium as the carrier. The components were separated using an Agilent DB-5MS column (30 m. 0.25 mm. 0.25 µm) with a temperature gradient ranging from 45°C to 180°C at a rate of 6°C min^-1^, followed by an increase to 300°C at a rate of 100°C min^-1^. To identify the compounds, the column outlet flow was connected to an Agilent 5973 quadrupole mass selective detector. The detector had the following settings: interface temperature of 270°C, quadrupole temperature of 150°C, source temperature of 230°C, and electron energy of 70 eV. The following parameters were used for peak integration: initial peak width 0.08, initial threshold 18.0, integrator 5000-25,000. The identity of each peak was determined by comparing its mass spectrum and retention time to authentic standards or spectra from reference libraries (NIST98 and Wiley 275) using MSD Chemstation. For compound quantification, the column outlet (with H2 as carrier gas) was connected to a flame ionization detector set at 300°C. The amount of each chemical was calculated by comparing its peak area to that of the internal standard.

### 2.6 Analysis of phenolic compounds by LC-MS/MS

We extracted and homogenized approximately 50 mg of fresh thyme in 1.0 mL of methanol containing 40 ng of D6-JA (HPC Standards GmbH, Germany) and 10 ng of trifluoro-methyl-cinnamic acid (Alfa Aesar) as internal standards. The samples were shaken on a horizontal shaker at room temperature for 10 min. The homogenate was blended for 30 minutes, then centrifuged at 13,000 rpm for 20 minutes at 4°C. The supernatant was then collected for analysis. The phenolic components in the leaf extracts were analyzed according to Ferlian et al. (2021) The Agilent 1260 Infinity II LC system (Agilent Technologies) was equipped with a Zorbax Eclipse XDB-C18 column (50 x 4.6 mm, 1.8 µm; Agilent Technologies), using aqueous formic acid (0.05% v/v) and acetonitrile as mobile phases A and B, respectively. The flow rate of the mobile phase was 1.1 mL min^-1^. The elution profile was as follows: 0-0.5 min, 5% B; 0.5-6.0 min, 5-37.4% B; 6.02-7.5 min, 80-100% B; 7.5-9.5 min, 100% B; and 9.52–12 min, 5% B. The column temperature was maintained at 20°C. The LC system was connected to a QTRAP 6500 tandem mass spectrometer (SCIEX) which had a turbo spray ion source that was set to negative ionization mode. The ion spray voltage was held at -4500 eV, and the turbo gas temperature was set to 650°C. The nebulizing gas was set to 60 psi, the curtain gas to 40 psi, the heating gas to 60 psi, and the collision gas to medium setting. Multiple reaction monitoring (MRM) was used for quantification: analyzing the analyte precursor ion (Q1) fragmentation into the product ion (Q3) (see **Supplementary Table S2**). Data gathering and processing were carried out using Analyst 1.6.3 software from Applied Biosystems’. Five compounds were absolutely quantified using D6-JA as an internal standard and the response factors indicated in **Supplementary Table S2**: 5-caffeoyl-quinic acid, 3- caffeoyl-quinic acid, 4-caffeoyl-quinic acid, and sulfo-jasmonic acid. Coumaric acid, caffeic acid, and ferulic acid were absolutely quantified with trifluoro-methyl-cinnamic acid as an internal standard with the response factors provided in **Supplementary Table S2**. All other substances were relatively quantified, and the results are expressed as peak area mg^-1^ of tissue (Ferlian et al., 2021).

### 2.7 Analysis of phytohormones by LC-MS/MS

Approximately 50 mg of fresh thyme tissue was extracted and homogenized in 1.0 mL methanol containing the following internal standards: 40 ng of D4-SA (Santa Cruz Biotechnology), 40 ng of D6-JA (HPC Standards GmbH), 40 ng of D6-ABA (Toronto Research Chemicals), and 8 ng D6-JA-Ile (HPC Standards GmbH). The samples were shaken on a horizontal shaker at room temperature for 10 min. The homogenate was blended for 30 min, then centrifuged at 13,000 rpm for 20 min at 4°C. The supernatant was collected for analysis. Phytohormone analysis was performed using LC-MS/MS (Heyer et al., 2018). This analysis used an Agilent 1260 series HPLC system (Agilent Technologies) and a QTRAP 6500 tandem mass spectrometer (SCIEX). Agilent Technologies’ Zorbax Eclipse XDB-C18 column (50 x 4.6 mm, 1.8 μm) was used for chromatographic separation. Water containing 0.05% formic acid and acetonitrile were used as mobile phases A and B, respectively. The elution profile was as follows: 0-0.5 min, 10% B; 0.5-4.0 min, 10-90% B; 4.0-4.02 min, 90-100% B; 4.02-4.55 min, 100% B; and 4.51-7.0 min, 10% B. The flow rate was held constant at 1.1 mL min^-1^, and the column temperature was kept at 25°C. The mass spectrometer was equipped with a turbo spray ion source operated in negative ionization mode. The ion spray voltage was set to -4,500 eV. The turbo gas temperature was set to 650°C. The nebulizing gas was set to 60 psi, the curtain gas at 40 psi, the heating gas at 60 psi, and the collision gas at “medium”. The mass spectrometer was operated in multiple reaction monitoring (MRM) mode. **Supplementary Table S3** contains information on the instrument parameters and response factors for quantification. Since both the D6-labeled JA and D6-labeled JA-Ile standards (HPC Standards GmbH) contained 40% of the corresponding D5-labeled compounds, the sum of the peak areas of the D5- and D6-labeled compounds was used for quantification.

### 2.8 Statistical analysis

The whole study was conducted as a factorial experiment using a completely randomized design with at least five biological replicates. The exact number of replicates is indicated in the particular figure legends. Data were tested for normality and homogeneity of variance using the Shapiro–Wilk and Levene’s tests, respectively, before ANOVA analysis. SAS version 9.4 (SAS Institute Inc.) was used to analyze all parameter data by ANOVA (CRD). Means were compared using the Least Significant Difference (LSD) *post-hoc* to detect significant differences (*p* ≤ 0.05). Additional analyses and figures were generated using Microsoft Excel 2019, JMP ver. 11, and Statgraphics ver. 16. Mean values are presented with their standard errors.

## 3 Results

### 3.1 Herbivore performance is different on different *Thymus* species

**Figure 1:**
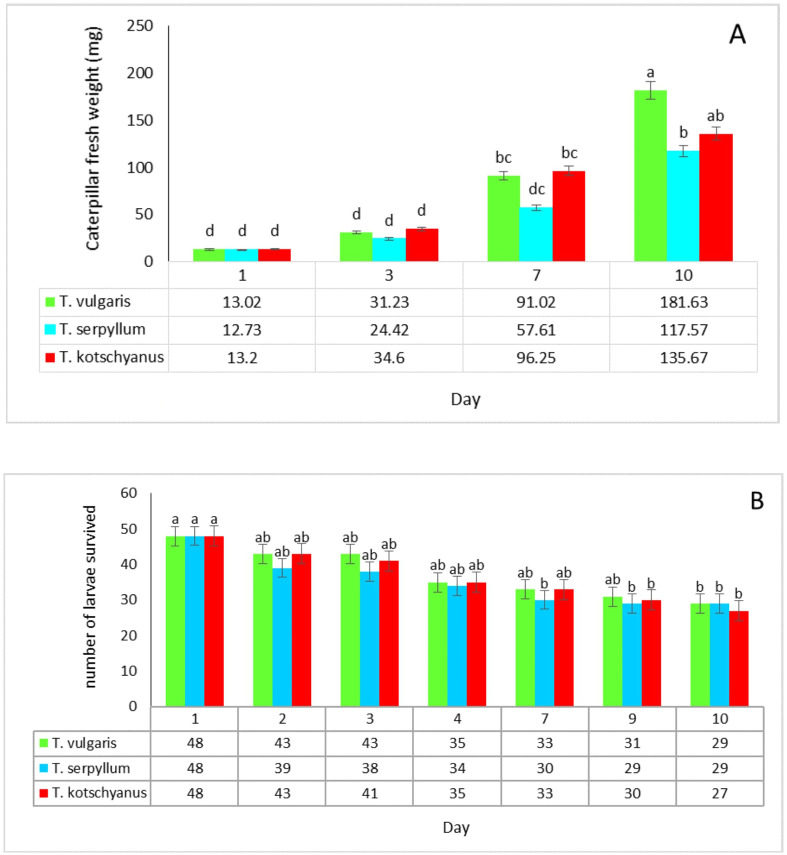
*Spodoptera littoralis* larvae performance and survival rates on three thyme species. **(A)** Caterpillar fresh weight on *T. vulgaris, T. serpyllum*, and *T. kotschyanus* was measured over a 10-day period under well-watered conditions. **(B)** Larval survival (number of surviving larvae). Data shown are mean ± SE, n = 8. Each treatment initially included 48 larvae (6 larvae per pot × 8 pots). Different letters indicate significant differences at *p* ≤ 0.05 (LSD test).

We investigated the performance of *S. littoralis*, measured as fresh weight and survival rate, over a period of 10 days for larvae feeding on three thyme species (*T. vulgaris, T. serpyllum* and *T. kotschyanus*), which differ not only in their drought tolerance but also in their content of terpenoids and phenolic compounds (Alipour et al., 2025). As shown in **Figure 1A**, larvae gained weight over time on all thyme species. No significant differences between the plant species were detected up to day 3. At day 7, larvae feeding on *T. serpyllum* showed reduced weight compared with the other thyme species. At day 10, there was a clear significant larval weight difference between larvae feeding on *T. serpyllum* (117.57 mg) and *T. vulgaris* (181.63 mg), while *T. kotschyanus* was in between (135.67 mg). Larval survival study (**Figure 1B**) started with 48 individuals per treatment (six larvae per pot × eight pots) and the number of larvae decreased gradually throughout the experiment on all thyme species. By day 7, survival had decreased to 33 living larvae for both *T. vulgaris*, and *T. kotschyanus*, while only 30 larvae survived on *T. serpyllum*, which was significantly different to day 1. At day 10, all survival rates on the three species were significantly reduced.

### 3.2 Drought stress and herbivory induced species-specific terpenoid patterns with highest levels observed after combined stress treatment

**Figure 2:**
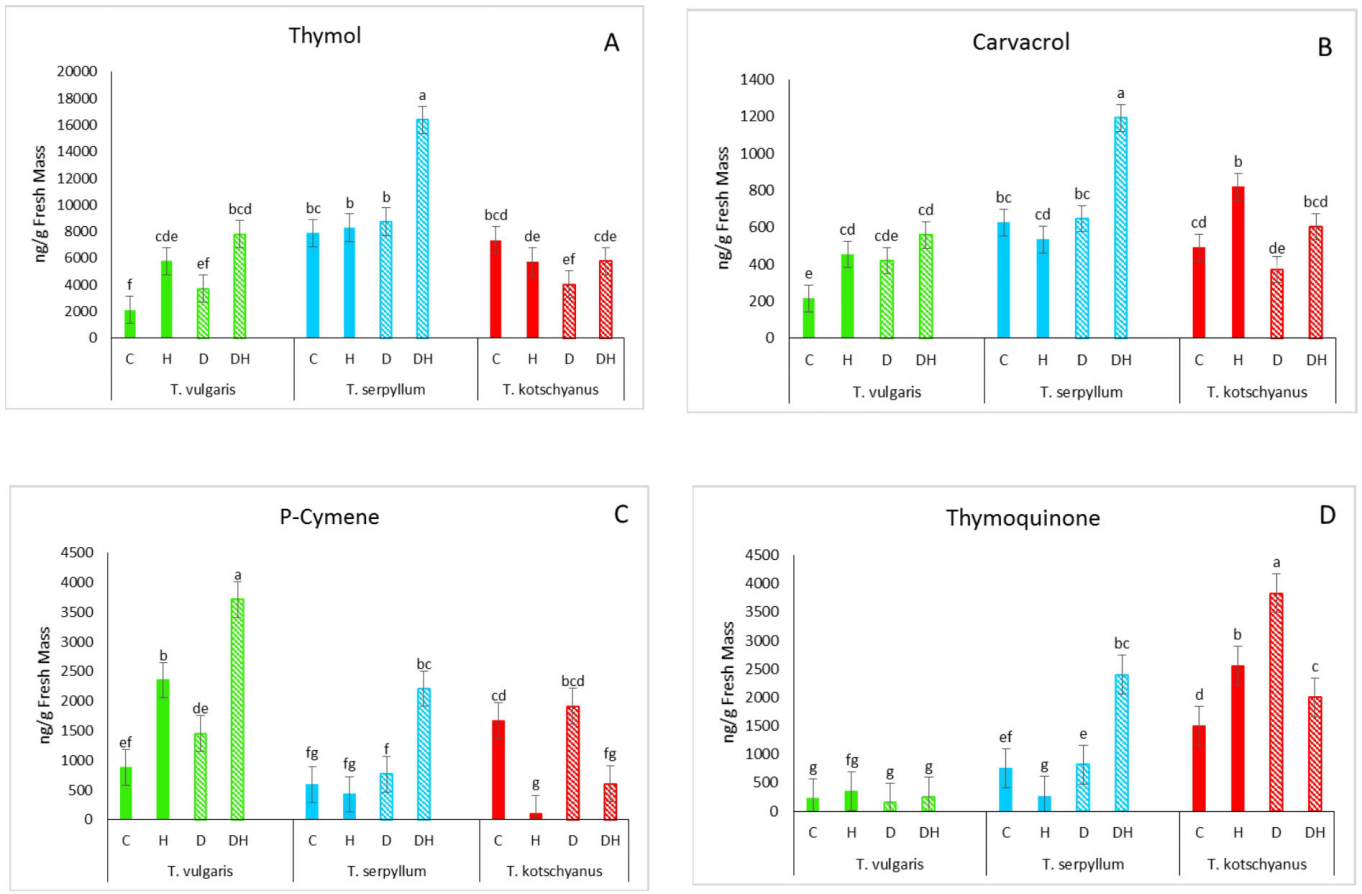
Species- and treatment specific enrichment of major terpenoid compounds. **(A)**, thymol; **(B)**, carvacrol; **(C)**, *p*-cymene; **(D)**, thymoquinone in *T. vulgaris* (green), *T. serpyllum* (blue) and *T. kotschyanus* (red) under different stress regimes: regular watering (filled bars), drought stress (striped bars). C, control; H, herbivory; D, drought; DH, drought × herbivory. Data shown are mean ± SE, n = 5, different letters indicate significant differences at *p* ≤ 0.05 (LSD test).

To determine the effects of drought and herbivory treatments on the major terpenoid compounds in the three thyme species, the amounts of thymol, carvacrol, *p*-cymene, and thymoquinone in the species’ leaves were analyzed (**Figure 2**). The three species exhibited substantial differences in the concentrations of the main terpenoids that accumulate in leaves and exhibited distinct responses to the treatments.

In *T. vulgaris*, drought stress increased the contents of thymol, *p*-cymene, and carvacrol. However, this effect was much more pronounced and significant after herbivore treatment. Highest contents were reached upon the combined stress, drought × herbivory. Thymoquinone level remained unaffected (**Figure 2**). In *T. serpyllum*, almost no significant changes of the analyzed terpenoid level were observed upon single stress treatments with the exception that thymoquinone was slightly reduced after herbivore treatment. Strikingly, the stress combination caused significant increases for all these compounds (**Figure 2**). In *T. kotschyanus* carvacrol content increased significantly due to herbivory in well-watered plants. The *p*-cymene levels were significantly decreased upon herbivore treatment. Thymoquinone was found to be the most responding compound. Both stress treatments caused a significant increase, while the combined stress attenuated these effects (**Figure 2**).

**Figure 3:**
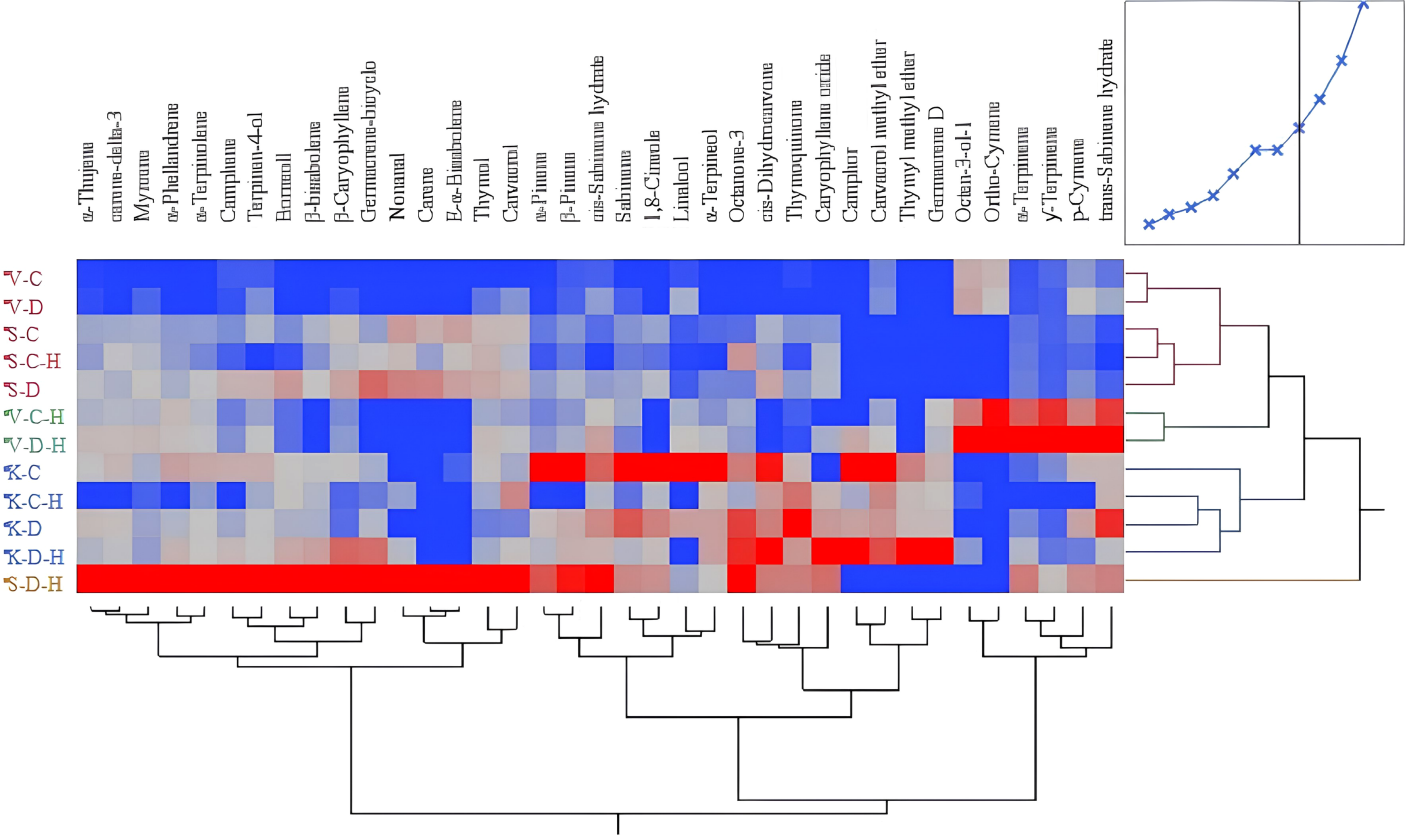
Hierarchically clustered heat map of various terpenoid compounds that accumulated in three different thyme species (V= *T. vulgaris*; S = *T. serpyllum*; K = *T. kotschyanus*) under different irrigation regimes (C, control or regular watering; D, drought stress) and herbivory (H). Color intensity indicates compound levels, with red representing higher abundance, gray intermediate abundance, and blue lower abundance.

In addition to focusing on the major terpenoids, we also compared the distribution of all terpenoid compounds in the three thyme species, both with and without stress treatment. A total of 37 compounds was identified in this experiment. Heat map revealed distinct clusters, with the heat map’s colors demonstrating that the species vary in the levels of terpenoids produced under both stress and non-stress conditions (**Figure 3**). Drought and herbivory treatment affected metabolites across the four clusters, but the patterns were different. *T. kotschyanus* terpenoid pattern clustered together, independent on the treatment. In contrast, herbivory led to a distinct clustering for *T. vulgaris*, while drought × herbivory combination induced a specific and unique profile in *T. serpyllum* (**Figure 3**).

Based on these analytical results, we examined the degree of variation among the 37 identified compounds using each terpenoid as a categorical variable and the proportion of compound content as a continuous variable. The variation in levels of the 37 compounds in the three thyme species under the different treatments varied from 0 to 20 µg g FM^-1^. Thymol was the highest and most variable component (0-16.4 µg g FM^-1^), followed by thymoquinone (0-3.8 µg g FM^-1^), *p*-cymene (0-3.7 µg g FM^-1^), and carvacrol (0-1.2 µg g FM^-1^) (**Supplementary Figure S1**). Overall, the results revealed a statistically significant relationship between water stress, herbivory, and the terpenoid profile of thyme species (**Supplementary Table S4**).

### 3.3 Drought and herbivory led to species-specific expression patterns of selected thymol/carvacrol biosynthetic pathway genes

**Figure 4:**
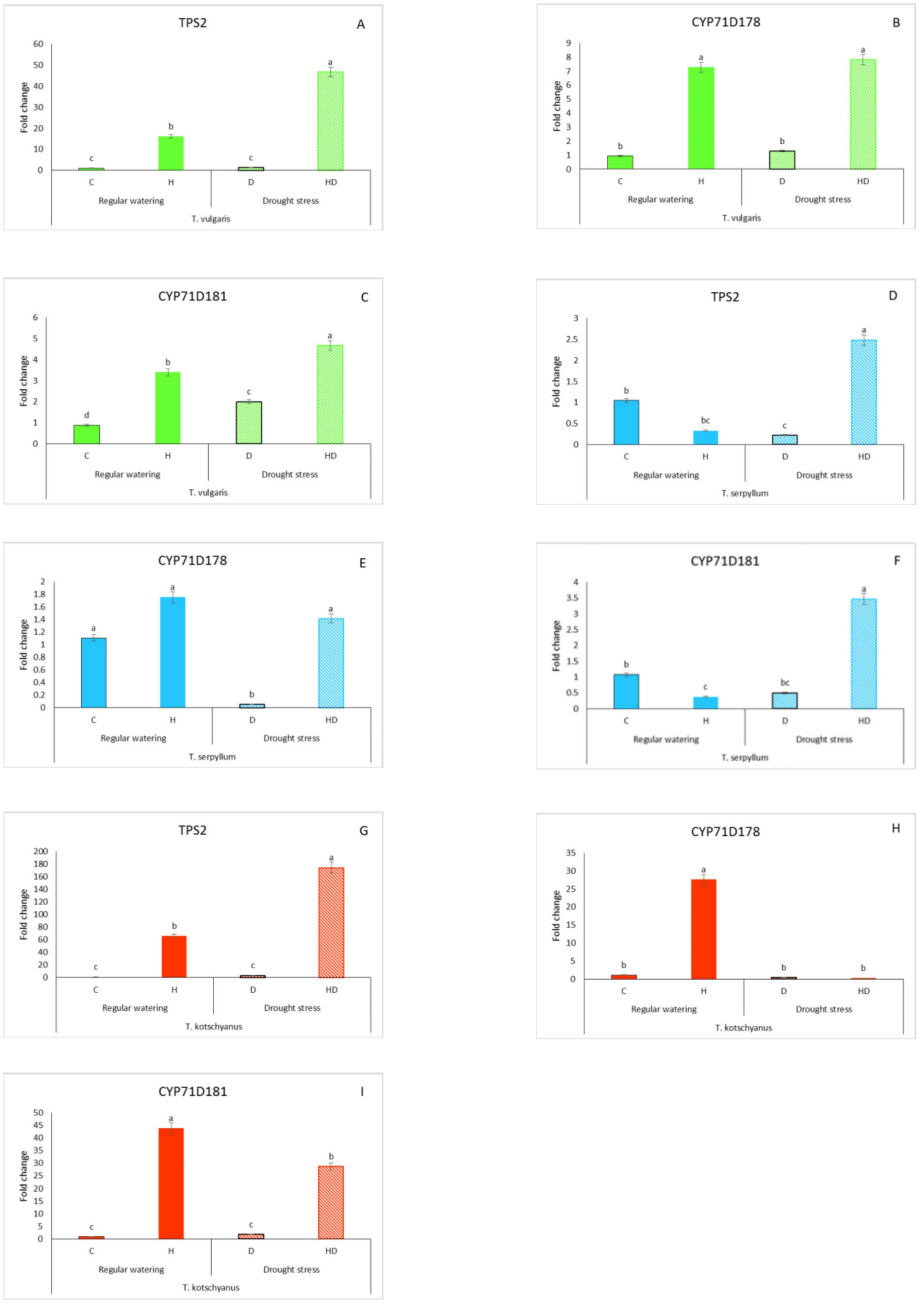
Species- and treatment-specific relative expression of terpenoid biosynthesis genes (*TPS2, CYP71D178*, and *CYP71D181*) in *T. vulgaris* (green, **A–C**), *T. serpyllum* (blue, **D–F**) and *T. kotschyanus* (red, **G–I**) leaves under different stress regimes: regular watering (filled bars), drought stress (striped bars). C, control; H, herbivory; D, drought; HD, drought × herbivory. Data shown are mean ± SE, n = 5, different letters indicate significant differences at *p* ≤ 0.05 (LSD test).

To examine the link between compound accumulation and gene expression in the leaves of the three thyme species, the relative gene expression of key biosynthetic enzymes of terpenoids such as *TPS2*, *CYP71D178*, and *CYP71D181*, respectively, was studied (**Figure 4**). In *T. vulgaris*, herbivory significantly induced the expression of all three genes investigated. Drought stress alone had almost no significant effect on the gene expression, except for *CYP71D181.* Significant differences were also observed in all tested genes in response to drought × herbivory, with values 46.8-, 7.82-, and 4.67- fold higher than those of the control and also higher than found for herbivory alone (**Figures 4A–C**). In *T. serpyllum*, the expression of *TPS2* and *CYP71D181* was slightly reduced by both herbivory and drought stress, whereas upon combined stress a significant increase of expression was detected. *CYP71D178* showed almost no significant change in expression except for a drought-induced reduction (**Figures 4D–F**). In *T. kotschyanus*, herbivory caused a high expression of all three genes, while drought alone had no effect. The combination of drought × herbivory caused the highest expression of *TPS2*, with values 174.1- fold higher than the control, a moderate but significant expression of *CYP71D181* and no expression of *CYP71D178* (**Figures 4G–I**).

Based on those data, Pearson’s correlation analysis was performed to determine any relationships between the major terpenoid components and relative transcript levels (**Supplementary Figure S2**). *CYP71D178* and *CYP71D181* exhibited the strongest correlation coefficient (*r* = 0.76**), followed by *TPS2* and *CYP71D181* (*r* = 0.74*). Among the terpenoids, thymol and carvacrol showed a strong correlation (*r* = 0.88**). The strongest negative correlation was found between *p*-cymene and *CYP71D181* (*r* = -0.40) (**Supplementary Figure S2**). Overall, we did not find a strong correlation between gene expression and the amount of major terpenoid compounds.

### 3.4 Drought stress and herbivory led to species-specific patterns of changes in phenolic compound composition and levels

In addition to terpenoids, the phenolic compounds in the leaves of *T. vulgaris*, *T. serpyllum*, and *T. kotschyanus* were examined under drought and herbivory. Sixteen compounds were identified in the analysis (**Supplementary Tables S5A–C**), including nine different phenolic acids (*p*-coumaric acid, caffeic acid, ferulic acid, rosmarinic acid, rosmarinic acid glucoside, quinic acid, and chlorogenic acids (5- chlorogenic acid (5CQA); 4- chlorogenic acid (4CQA); and 3- chlorogenic acid (3CQA)), citric acid, three flavonoids (apigenin-6,8-di-C-glucoside, apigenin-glucuronide, and luteolin-glucuronide). Three unknown compounds were also detected (555-359, 307-263, and 399-161). Both herbivory and drought treatments significantly altered the polyphenol content in the thyme species. Rosmarinic acid exhibited the largest peak area of the phenolic components measured in all studied thyme species (**Supplementary Tables S5A–C**).

The levels of phenolic compounds were primarily determined by species. *T. vulgaris* is characterized by high levels of rosmarinic acid, luteolin-glucoronide, apigenin-glucoronide, and the unknown compounds 555-359, 399–161 and 307-263, while the two other species tended to be more similar to each other in their phenolic profiles (**Supplementary Tables S5**). Herbivory induced higher levels of phenylpropanoids, i.e. coumaric acid, caffeic acid and ferulic acid in all species, though absolute levels differed. In combination with drought, the amounts of these compounds further increased except coumaric acid in *T. vulgaris*. Interestingly, also rosmarinic acid, apigenin-6-8-di-C-glucose, rosmarinic acid glucoside and chlorogenic acids significantly increased after the combined stress of drought × herbivory (**Supplementary Tables S5B, C**). Only citric acid levels significantly declined in all three species when exposed to the combination of drought stress and herbivory.

**Figure 5:**
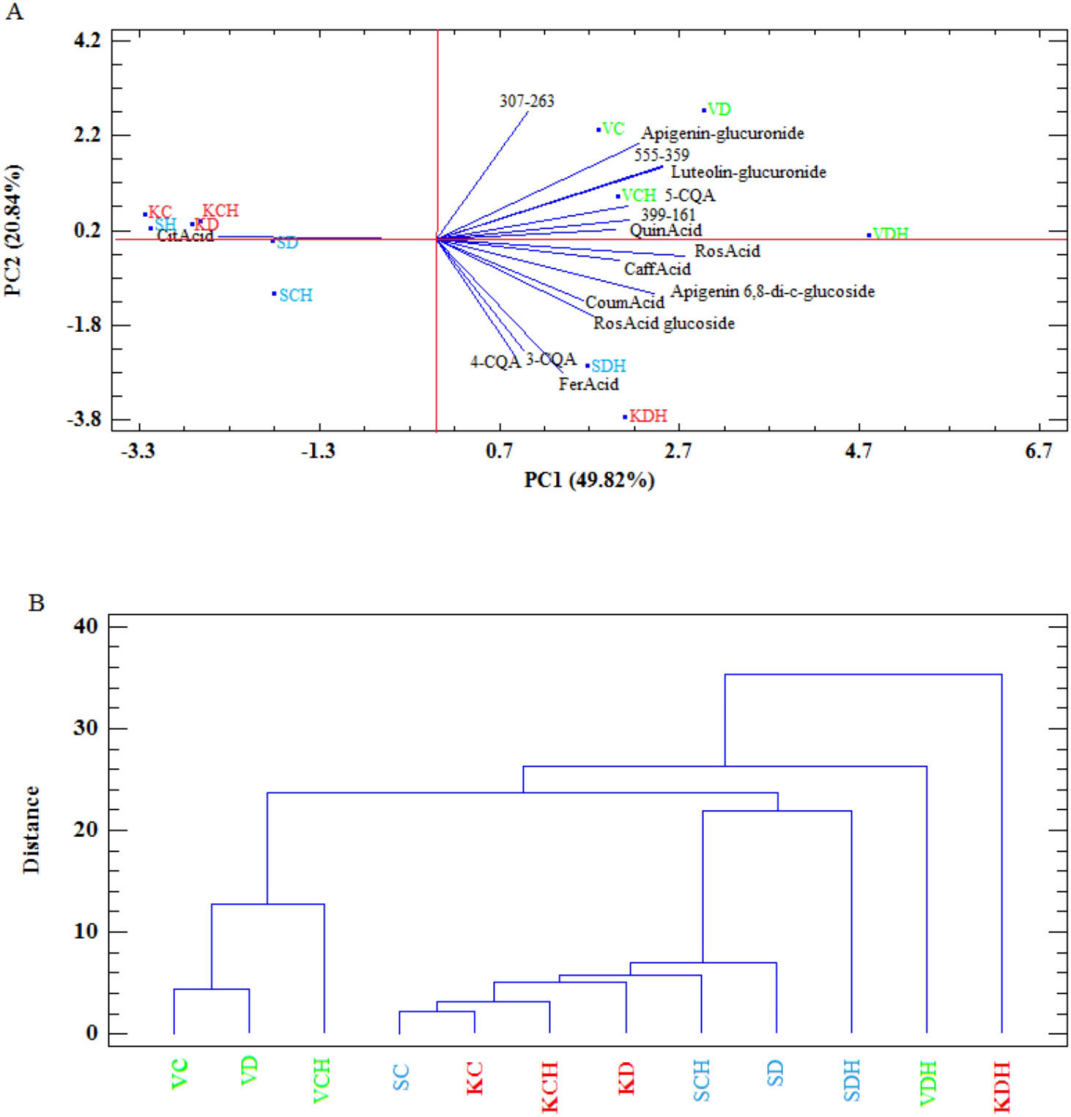
Principal component analysis (PCA) **(A)** and Cluster Analysis (CA) **(B)** for stress-related polyphenols in thyme species (V= *T. vulgaris* (green), S= *T. serpyllum* (blue), K= *T. kotschyanus* (red)) treated with different irrigation regimes (C, control or regular watering; D, drought stress) and herbivory (H) treatment, point labels: number of complete cases; vectors: data variables.

A principal component analysis (PCA; **Figure 5A**; **Supplementary Table S7**) and a cluster analysis (**Figure 5B**) corroborated the trends observed for the individual compounds. PC1 (49.82%) and PC2 (20.84%) together explained 70.66% of the total variance in the phenolic profiles of the three thyme species. Drought × herbivory stressed plants were strongly associated with higher levels of rosmarinic acid, chlorogenic acids (3-CQA, 4-CQA, and 5-CQA), luteolin-glucuronide, and apigenin-glucuronide. Control, drought and herbivory-stressed samples of *T. kotschyanus* and *T. serpyllum* showed lower phenolic contents, with citric acid predominating, while *T. vulgaris* was separately associated with the remaining phenolic compounds analyzed (**Figure 5A**). A cluster analysis also supported these findings (**Figure 5B**).

### 3.5 Phytohormone levels increase after herbivory, combined herbivore and drought stress

**Figure 6:**
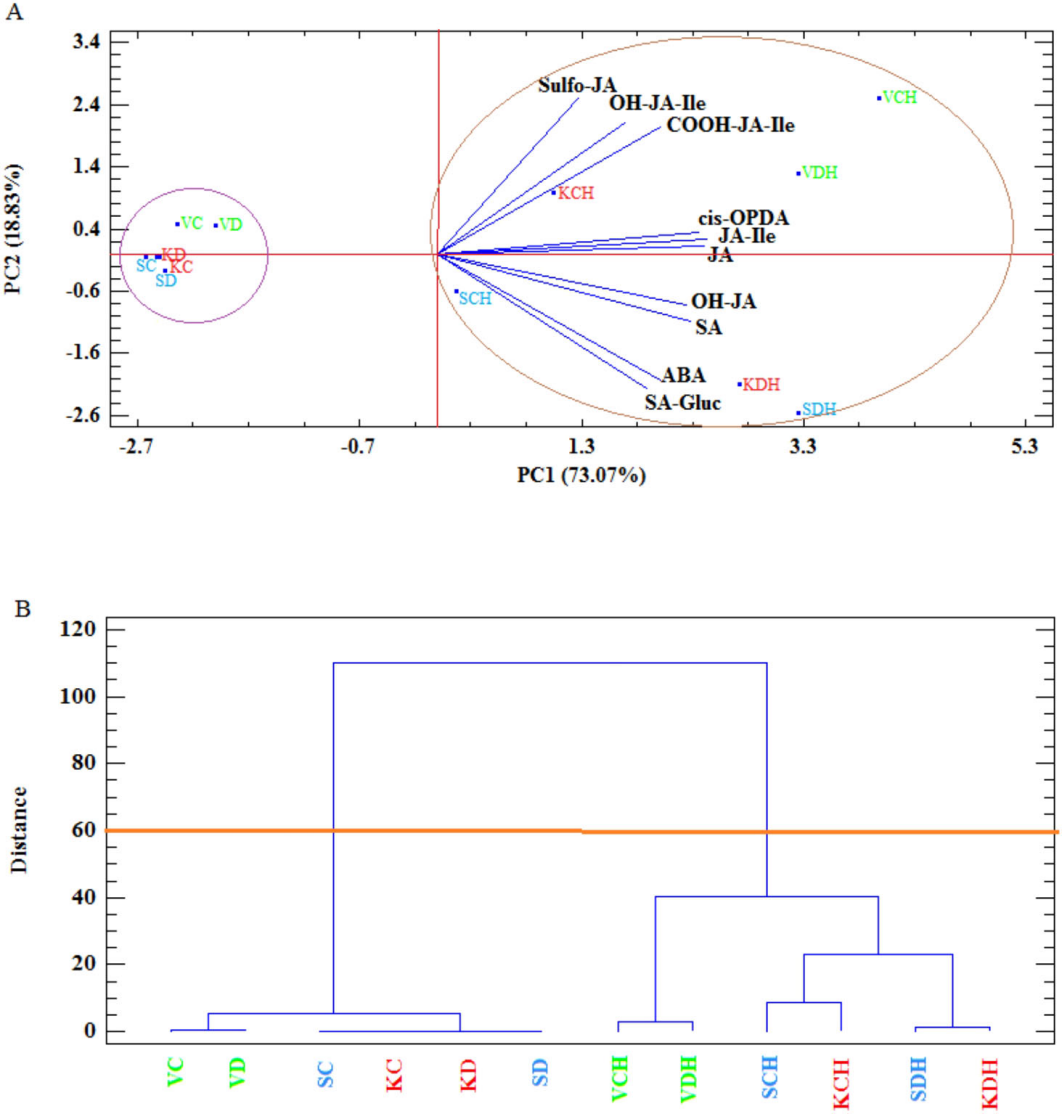
Principal component analysis (PCA) **(A)** and Cluster Analysis (CA) **(B)** for stress-related phytohormones in thyme species (V= *T. vulgaris* (green), S= *T. serpyllum* (blue), K= *T. kotschyanus* (red)) treated with different irrigation regimes (C, control or regular watering; D, drought stress) and herbivory (H). SA (salicylic acid), SA-Gluc (salicylic acid-b-D-glucoside), cis-OPDA (12-oxophytodienoic acid), JA (jasmonic acid), JA-Ile (jasmonoyl-isoleucine conjugate), OH-JA-Ile (hydroxyjasmonoyl-isoleucine conjugate), OH-JA (hydroxyjasmonic acid), COOH-JA-Ile (carboxy jasmonoyl-isoleucine conjugate), Sulfo-JA (sulfated jasmonic acid), ABA (abscisic acid); point labels: number of complete cases, vectors: data variables.

Phytohormones mediate stress responses in plants. As expected, *cis*-OPDA, JA and JA-conjugates significantly increased in all herbivore-treated samples, though absolute levels partially differed among the species. Also, the response to additional drought stress was different among the species. While in *T. vulgaris* drought × herbivory induced significantly lower or similar levels of JA and JA-related compounds, in *T. serpyllum* these levels mostly increased, in particular JA, JA-Ile and OH-JA. *T. kotschyanus* showed a similar, but less pronounced picture. The sulfo-JA levels were highest in *T. vulgaris*, in *T. kotschyanus* about 5 to 10 times lower and drastically lower in *T. serpyllum*. Nevertheless, the treatments affected the sulfo-JA levels (**Supplementary Table S6**). Interestingly, not only JA and JA-conjugate levels increased after herbivory but also SA and ABA concentrations, with highest concentrations after combined drought × herbivory stress. Drought treatment only had no or a very minor effect on phytohormone levels (**Supplementary Table S6**). In accordance with these findings at the individual compound level, PCA revealed that PC1 (73.07%) and PC2 (18.53%) together explained over 91% of the total variance, and clearly separated the treatments. The control and drought treatments without herbivory clustered on the left side of PC1, while drought × herbivory and herbivory alone grouped on the right side of PC1 and were associated with higher levels of JA, JA-Ile, *cis*-OPDA, SA, ABA and related metabolites (**Figure 6A**; **Supplementary Table S8**). Similarly, the cluster analysis clearly grouped all control and drought treated samples in one cluster independent of the species, while the herbivory and drought × herbivory treated samples of *T. vulgaris* clustered together, as well as *T. kotschyanus* and *T. serpyllum* after herbivory and after combined stress, respectively (**Figure 6B**).

## 4 Discussion

Herbivory and drought are two key factors that threaten agricultural production, particularly in semi-arid areas. This study aimed to determine how drought, herbivory, and their combination affect the secondary metabolism and hormonal regulation in three thyme species differing in drought tolerance. Thus, we analyzed the changes in the content of terpenoids, phenolics, and phytohormones under individual and combined drought stress and insect herbivory.

Up to now, only very few studies have been conducted addressing the performance of feeding insects on thyme species. We employed the generalist *Spodoptera littoralis* as serious pest in agriculture to perform feeding assays on the three thyme species. Over a time period of 10 days, *S. littoralis* larvae gained the most biomass on *T. vulgaris* and significantly less biomass when feeding on *T. serpyllum* suggesting for this latter species a greater resistance to herbivory (**Figure 1B**). Nevertheless, the significantly reduced survival rate of the larvae after 10 days (about 60%) was similar on all three thyme species indicating their general toxic properties (**Figure 1B**).

We speculate that the chemical composition may play a crucial role in the reduced larval weight gain observed on *T. serpyllum* compared with *T vulgaris*. Thymol, carvacrol, or *p*-cymene, the major terpenoid compounds of thyme, are known to influence herbivore feeding behavior and have known insecticidal and repellent properties (Linhart and Thompson, 1999; Sedy and Koschier, 2003; Agliassa and Maffei, 2018; López et al., 2021). For example, thymol and carvacrol were toxic against brown planthopper (*Nilaparvata lugens*), a rice pest, with an LD_50_ as low as 600 μg·L^-1^ in fumigant bioassays (Peng et al., 2025). Thymol and carvacrol concentrations were per se significantly higher in *T. serpyllum* than in *T. vulgaris* and following herbivory, their concentrations further increased in *T. serpyllum* to higher level compared with *T. vulgaris* (**Figure 2**). This may have contributed to the reduced weight gain of larvae feeding on *T. serpyllum*. As the overall level of *p*-cymene is highest in *T. vulgaris* compared with the two other thyme species, it seems unlikely that this particular compound has a strong effect on the larvae’s performance. In addition, thymoquinone was highest in *T. kotschyanus* suggesting that this terpenoid neither has a positive nor negative impact on the insects because larval weight gain was between *T. vulgaris* and *T. serpyllum* (**Figure 1**). However, a previous study suggested that the carvacrol precursor *γ*-terpinene is more toxic to *S. littoralis* than carvacrol or *p*-cymene (Agliassa and Maffei, 2018); but in the present study, *γ*-terpinene was mainly enriched in *T. vulgaris*, and not in the more resistant species *T. serpyllum* (**Figure 3**). Thus, it is conceivable that carvacrol, *p*-cymene, thymol, and other terpenoids exhibit synergistic toxic effects with other monoterpenes (Tak and Isman, 2017). The thyme species not only differed in the accumulation of the major terpenoids, but also in other terpenoids as well as in phenolic compounds’ contents. Thus, phenolic compounds may play an additional role in the reduced caterpillar growth on *T. serpyllum.* Additionally, other factors, such as differences in the nutritional value may have affected caterpillar growth.

One seemingly clear result was that drought stress had only minor effects on the measured compounds (**Figures 3**, **5**). Thymoquinone in *T. kotchyanus* appears to be an exception (**Figure 2**). In contrast, herbivory caused more changes in the profile of both terpenoids and phenolic compounds than drought stress (**Figures 2**, **3**, **5**). However, the pattern of compounds obtained remained species-specific rather than treatment-specific. However, the results regarding drought stress should not be overinterpreted or underestimated. It has already been shown that negligible responses to individual stressors in stress combinations or to subsequent stress had a major effect, i.e. the plants were primed (Zandalinas et al., 2021).

Besides investigating the plant responses to drought stress and herbivory, a main goal of this study was to find out how plant metabolism is affected by combined drought × herbivory stress, as thyme is an important crop and its value depends on the amount and composition of essential oils. Consistent with previous reports (see the review by Sahu and Giri, 2025), the largest metabolic changes were indeed observed after combined drought × herbivory stress. Strikingly, also these changes were largely species-specific. Combined stress increased the concentration of thymol, carvacrol and *p*-cymene in *T. vulgaris* and *T. serpyllum*, while in *T. kotschyanus* the levels of *p*-cymene and thymoquinone significantly decreased compared to drought stress alone and differ from sole herbivory stress (**Figure 2**). In addition to these major terpenoid compounds, the species-specific enrichment of other terpenoids is notable. The drought-sensitive species *T. serpyllum* showed the highest number of different enriched terpenoids after combined stress, with many changes compared to individual stress treatments (**Figure 3**). In contrast, the changes in the terpenoid profile of *T. kotschyanus* were less pronounced across all treatments; this could reflect its higher drought resistance (**Figure 3**). *T. vulgaris* demonstrated a strong induction of some specific terpenoids in response to herbivory, and accumulation is further slightly enhanced by additional drought treatment (**Figure 3**). The enriched metabolites in *T. vulgaris* were clearly distinct from those enriched in *T. serpyllum*. This specific response to herbivory may reflect its higher susceptibility to herbivory, and could be part of an adaptive response to reduce herbivory and oxidative damage (Gaude and Jalmi, 2025).

The species-specific increase in different terpenoid compounds after various forms of stress aligns at least in part with the distinct expression patterns of *TPS2*, *CYP71D181*, and *CYP71D178* among the three thyme species. Although in all three thyme species the high accumulation of thymol, carvacrol, and *p*-cymene following combined stress treatment mirrors mostly the observed increase in expression patterns of the genes analyzed, there are exceptions such as *CYP71D178* in *T. kotschyanus* (**Figures 2**, **4**) and the upregulation of all three genes following herbivory alone in *T. vulgaris* and *T. kotschyanus* but their downregulation in *T. serpyllum* (**Figure 4**). These unexpected gene expression pattern maybe further investigated in future. Nevertheless, also gene expression responses are species-specific rather than depending mainly on the particular stress.

Phytohormones interact with each other and then trigger signaling processes that are complex and dynamic (Salvi et al., 2021). Thus, they cannot be considered in isolation. For example, JA mainly regulate defense against herbivory, while ABA accumulates and regulates drought stress responses but both have been found to function in concert. They share molecular targets such as transcription factors, and common genetic networks. In contrast, JA and SA often act antagonistically. Drought stress upregulates the biosynthesis of JA and inhibits SA biosynthesis. Lesser amount of cellular SA triggers increased production of ABA. For the measured phytohormones, it seems clear that herbivory makes the difference rather than drought stress (**Figure 6**). This is somewhat surprising because here the response is treatment- and not species-specific. All three thyme species displayed elevated levels of JA, SA, and ABA when exposed to herbivory, as well as to combined drought × herbivory stress (**Supplementary Table S6**). It is well known that JA and ABA interact in stress-induced signal transduction (Rosa-Diaz et al., 2024; Sahu and Giri, 2025). Not only has it been described that these two phytohormones can result in the same response, but it has also been shown that both signaling pathways may directly affect each other. Plant resistance to herbivory can be reduced in the absence of ABA. For instance, ABA is required to regulate JA-dependent defense pathways in *Arabidopsis thaliana* following caterpillar feeding (Vos et al., 2013), and ABA-deficient tomato plants are more susceptible to *Spodoptera exigua* feeding (Thaler and Bostock, 2004). Furthermore, JA and ABA jointly regulate the biosynthesis of the terpenoid artemisin in *Artemisia annua* (Jing et al., 2009). The effect of the two phytohormones on compound accumulation may be either synergistic or antagonistic. In all three species analyzed in the present study, both phytohormones were induced by herbivory, while the terpenoid pattern differed depending on the water regime. This suggests that they have a minor regulatory role on this compound class in thyme. In contrast, phytohormone levels correlated with the accumulation of phenolic compounds and may have a regulatory function here. Interestingly, not only are ABA and JA induced by herbivory in all three species, but SA is also induced, with higher values observed when additional drought stress is applied (**Supplementary Table S6**). This is rather unexpected, given that SA and JA often act antagonistically (Thaler et al., 2012). However, joint feeding by the Colorado potato beetle - but not *S. exigua* feeding - and drought stress also induced JA and SA in *Solanum dulcamara*. This led to higher pathogen resistance after beetle feeding (Nguyen et al., 2018).

The reason for the dominant herbivory-related responses may lie in the experimental design, where herbivory was applied as second stress factor and plants were harvested directly after this particular stress application. Thus, the sequence in which the two stresses were applied may be crucial in determining the plant response. It is known that the expression of the related plant responses to sequential and combined stress depends on the strength and order of the stress events (Anwar et al., 2021; Zandalinas et al., 2021; Jiang et al., 2025). However, given that a plant is challenged with sequential stress combination, even if the initial stress is low, it might induce direct or priming effects, which can influence the responses to the additional stress (Jiang et al., 2025). Such responses are more than additive effects and were observed in our study for the drought × herbivory approach by the significant increase of terpenoids in *T. serpyllum* and in part in *T. vulgaris* but not in *T. kotchyanus* (**Figure 2**) and were also visible in the terpenoid pattern, in particular for *T. serpyllum* and *T. kotchyanus* (**Figure 3**). Concerning the polyphenol contents, all three thyme species showed an increase due to drought × herbivory stress, though with varying degrees (**Figure 5**).

In summary, this is the first integrative study linking metabolomic, hormonal, and transcriptional responses under combined drought–herbivory stress in *Thymus* species. The study demonstrates that the interplay between drought × herbivory has species-specific effects on secondary metabolite accumulation in *Thymus* spp., while phytohormones show treatment-specific, i.e. herbivory-specific responses. Combined stress generally amplifies both phytohormonal responses, particularly those of JA and ABA, as well as the biosynthesis of key defensive metabolites, including thymol, carvacrol, rosmarinic acid and chlorogenic acid. However, the induced terpenoid and phenolics profiles were highly species-specific and could be related to the differences in drought and herbivore-tolerance of the three thyme species. In future studies, we plan to investigate in more detail how the combined stress is affecting signaling pathways in order to better understand defense responses, and whether joint drought × herbivory stress can also alter pathogen resistance in the plants. The latter has enormous biotechnological, agricultural, and ecological impact.

## Data Availability

The original contributions presented in the study are included in the article/**Supplementary Material**. Further inquiries can be directed to the corresponding authors.
