# Supplementary material for: Drought, herbivory and combined stress caused treatment-specific changes in phytohormones, but species-specific changes in secondary metabolite profiles in thyme
Source: Front Plant Sci. 2025 Dec 1;16:1711338. doi: 10.3389/fpls.2025.1711338 (PMC12702949; doi:10.3389/fpls.2025.1711338)
Supplement: Supplementary file 1 [file DataSheet1.docx]

***Supplementary Material***

1. **Supplementary Tables and Figures**
   1. **Supplementary Tables**

**Supplementary Table S1.** Primers used for qRT-PCR analysis.

| Real-Time Primers | Sequences (5´ to 3´) | Tm°C | Reference |
| --- | --- | --- | --- |
| *Ef1 F*  *Ef1 R* | AGATCGGAAATGGTTATGCTC  GACCTCCTGTCAATCTTCGT | 55.9  57.3 | Ashrafi *et al*., 2022 |
| *TPS2 F*  *TPS2 R* | AACCTCGCCGAGAAACTCCC  AGCTGCAGTTCGTCGAGTGT | 61.4  59.4 | Tohidi *et al*., 2020 |
| *CYP71D178 F*  *CYP71D178 R* | CAAGGAATGACTGCTGCTGAC  TTGGATTGTGGATTGTTGGAACC | 59.8  58.9 | Crocoll, 2011 |
| *CYP71D181 F*  *CYP71D181 R* | TACTGGAAAGACCCCGACAC  CGAACGGGATTAACTCGAAA | 59.4  55.3 | Crocoll, 2011 |

References:

Ashrafi, M., Azimi-Moqadam, M. R., MohseniFard, E., Shekari, F., Jafary, H., Moradi, P., et al. (2022). Physiological and molecular aspects of two *Thymus* species differently sensitive to drought stress. *BioTech*. 11(2), 8. doi: org/10.3390/biotech11020008.

Crocoll, C. (2011). Biosynthesis of the phenolic monoterpenes, thymol and carvacrol, by terpene synthases and cytochrome P450s in oregano and thyme (Doctoral dissertation, Jena, Friedrich-Schiller-Universität Jena, Diss., 2011).

Tohidi, B., Rahimmalek, M., Arzani, A., and Trindade, H. (2020). Sequencing and variation of terpene synthase gene (*TPS2*) as the major gene in biosynthesis of thymol in different *Thymus* species. *Phytochemistry* 169, 112126. doi: org/10.1016/j.phytochem.2019.112126.

**Supplementary Table S2**. Details of analysis of phenolic compounds by LC-MS/MS [HPLC 1260 (Agilent Technologies)-QTRAP6500 (SCIEX)] in negative ionization mode

| Q1 | Q3 | RT (min) | Compound | Internal std | RF | DP | EP | CE | CXP |
| --- | --- | --- | --- | --- | --- | --- | --- | --- | --- |
| 353.0 | 190.9 | 3.65 | 5-caffeoyl-quinic acid | D6-JA | 1.15 | -20 | -4 | -22 | -4 |
| 353.0 | 179.1 | 3.00 | 3-caffeoyl-quinic acid | D6-JA | 0.89 | -20 | -4 | -22 | -4 |
| 353.1 | 173.0 | 3.80 | 4-caffeoyl-quinic acid | D6-JA | 0.81 | -20 | -4 | -22 | -4 |
| 305.0 | 97.0 | 4.00 | Sulfo-Jasmonic acid | D6-JA | 6.00 | -20 | -5 | -55 | -2 |
| 163.0 | 118.9 | 4.70 | coumaric acid | TriFluoro-methyl-cinnamic acid | 2.56 | -20 | -8 | -20 | -5 |
| 179.0 | 134.9 | 4.00 | caffeic acid | TriFluoro-methyl-cinnamic acid | 1.62 | -20 | -8 | -22 | -5 |
| 193.1 | 133.9 | 5.00 | ferulic acid | TriFluoro-methyl-cinnamic acid | 7.77 | -20 | -8 | -22 | -5 |
| 359.0 | 161.0 | 5.70 | rosmarinic acid |  |  | -20 | -8 | -25 | -5 |
| 493.0 | 295.0 | 5.90 | salvianolic acid-295-1 |  |  | -20 | -8 | -25 | -5 |
| 493.0 | 295.0 | 6.10 | salvianolic acid-295-2 |  |  | -20 | -8 | -25 | -5 |
| 593.0 | 473.0 | 4.00 | apigenin-6,8-di-C-glucoside |  |  | -20 | -8 | -25 | -5 |
| 521.0 | 359.0 | 5.00 | rosmarinic acid glucoside |  |  | -20 | -8 | -25 | -5 |
| 461.0 | 285.0 | 5.20 | luteolin-glucuronide |  |  | -20 | -8 | -25 | -5 |
| 445.0 | 269.0 | 5.50 | apigenin-glucuronide |  |  | -20 | -8 | -25 | -5 |
| 537.0 | 493.0 | 6.00 | lithospermic acid A |  |  | -20 | -8 | -25 | -5 |
| 555.0 | 359.0 | 5.70 | 555-359 |  |  | -20 | -8 | -25 | -5 |
| 307.0 | 263.0 | 1.90 | 307-263 |  |  | -20 | -8 | -25 | -5 |
| 399.0 | 161.0 | 4.40 | 399-161 |  |  | -20 | -8 | -25 | -5 |
| 301.0 | 93.0 | 6.80 | quercetin |  |  | -20 | -8 | -46 | -5 |
| 191.0 | 111.0 | 0.70 | citric acid |  |  | -20 | -8 | -18 | -5 |
| 191.0 | 85.0 | 0.50 | quinic acid |  |  | -20 | -8 | -28 | -5 |
| 215.1 | 171.1 | 7.30 | TriFluoro-methyl-cinnamic acid |  |  | -20 | -8 | -18 | -4 |
| 215.0 | 59.0 | 7.20 | D6-JA |  |  | -20 | -9 | -24 | -2 |

**Supplementary Table S3**. Details of analysis of phytohormones by LC-MS/MS [HPLC 1260 (Agilent Technologies)-QTRAP6500 (SCIEX)] in negative ionization mode

| Q1 | Q3 | RT (min) | Compound | Internal std | RF | DP | EP | CE | CXP |
| --- | --- | --- | --- | --- | --- | --- | --- | --- | --- |
| 136.93 | 93.00 | 3.3 | SA | D4-SA | 1.0 | -20 | -8 | -24 | -7 |
| 263.00 | 153.20 | 3.4 | ABA | D6-ABA | 1.0 | -20 | -12 | -22 | -2 |
| 209.07 | 59.00 | 3.6 | JA | D6-JA | 1.0 | -20 | -9 | -24 | -2 |
| 322.19 | 130.10 | 3.9 | JA-Ile | D6-JA-Ile | 1.0 | -50 | -4.5 | -30 | -4 |
| 290.90 | 165.10 | 4.6 | OPDA | D6-JA | 1.0 | -20 | -12 | -24 | -2 |
| 338.10 | 130.10 | 3 | OH-JA-Ile | D6-JA-Ile | 1.0 | -50 | -4.5 | -30 | -4 |
| 225.10 | 59.00 | 2.6 | OH-JA | D6-JA | 1.0 | -20 | -9 | -24 | -2 |
| 352.10 | 130.10 | 3 | COOH-JA-Ile | D6-JA-Ile | 1.0 | -50 | -4.5 | -30 | -4 |
| 140.93 | 97.00 | 3.3 | D4-SA |  |  | -20 | -8 | -24 | -7 |
| 269.00 | 159.20 | 3.4 | D6-ABA |  |  | -20 | -12 | -22 | -2 |
| 215.00 | 59.00 | 3.6 | D6-JA |  |  | -20 | -9 | -24 | -2 |
| 214.00 | 59.00 | 3.6 | D5-JA |  |  | -20 | -9 | -24 | -2 |
| 328.19 | 130.10 | 3.9 | D6-JA-Ile |  |  | -50 | -4.5 | -30 | -4 |
| 327.19 | 130.10 | 3.9 | D5-JA-Ile |  |  | -50 | -4.5 | -30 | -4 |

**Supplementary Table S4**. Analysis of variance of the data for the main terpenoids found in thyme species after drought and herbivory treatments.

|  | | mean squares | | | |
| --- | --- | --- | --- | --- | --- |
|  | df | Thymol | Carvacrol | *p*-Cymene | Thymoquinone |
| Species | 2 | 172692592.1** | 570276.68** | 7564929.18** | 25458771.66** |
| Drought | 1 | 35872803.9** | 176753.49** | 8943698.09** | 5999056.39** |
| Herbivory | 1 | 105928809.3** | 808250.83** | 1911444.52** | 119109.09ns |
| Species × Drought | 2 | 47078086.1** | 327016.97** | 593547.05** | 1993939.07** |
| Species × Herbivory | 2 | 25153110.1** | 10832.373ns | 13970072.71** | 1086414.41** |
| Drought × Herbivory | 1 | 50540772.9** | 82041.455* | 2943832.41** | 294374.89** |
| Species × Drought × Herbivory | 2 | 14662379.7** | 224938.617** | 579250.26** | 7769566.76** |
| Error | 48 | 950202.2 | 11738.035 | 74792.60 | 36326.35 |

ns, * and **: No significant and significant at *p* ≤ 0.05 and *p* ≤ 0.01, respectively.

**Supplementary Table S5A.** Phenolics content (ng g FW^-1^) in thyme species after drought and herbivory treatments.

| Specie | Irrigation regimes (%fc) | Herbivory | Coumaric acid | Caffeic acid | Ferulic acid | chlorogenic acid (5CQA) | chlorogenic acid (4CQA) | chlorogenic acid (3CQA) |
| --- | --- | --- | --- | --- | --- | --- | --- | --- |
| *T. vulgaris* | Regular watering | absent | 846 def | 55110 abc | 274 f | 15764 b | 22856 bc | 6787 b |
|  |  | presence | 3937 a | 76162 a | 1568 de | 4349 c | 26760 bc | 7645 b |
|  | Drought stress | absent | 617 ef | 46675 bcd | 316 f | 29935 a | 19689 bc | 7975 b |
|  |  | presence | 2545 b | 57391 abc | 2803 bc | 12443 b | 28432 bc | 11633 b |
| *T. serpyllum* | Regular watering | absent | 438 ef | 38602 cde | 375 f | 275 c | 7338 bc | 1353 b |
|  |  | presence | 1796 bcd | 51075 a-d | 1822 cd | 1040 c | 20204 bc | 5779 b |
|  | Drought stress | absent | 723 ef | 33833 c-f | 656 def | 942 c | 28301 bc | 3978 b |
|  |  | presence | 2678 b | 73173 ab | 4104 a | 4148 c | 59264 b | 11977 b |
| *T. kotschyanus* | Regular watering | absent | 496 ef | 17803 ef | 459 ef | 255 c | 2870 c | 438 b |
|  |  | presence | 1388 cde | 23789 edf | 1244 def | 583 c | 9197 bc | 2880 b |
|  | Drought stress | absent | 269 f | 9559 f | 557 ef | 123 c | 1061 c | 246 b |
|  |  | presence | 2042 bc | 42340 cde | 3039 ab | 19215 b | 478111 a | 114482 a |

**Supplementary Table S5B.** Phenolics content (peak area mg FW^-1^) in thyme after drought and herbivory treatments.

| Specie | Irrigation regimes (%fc) | Herbivory | Rosmarinic acid | Quinic acid | Rosmarinic acid glucoside | Citric acid | Apigenin-6,8-di-C-glucoside |
| --- | --- | --- | --- | --- | --- | --- | --- |
| *T. vulgaris* | Regular watering | absent | 15699916 ab | 1632644 abc | 284390 cde | 1146864 cd | 17460 cde |
|  |  | presence | 14735491 b | 2074357 ab | 144075 de | 1142095 cd | 11415 c-f |
|  | Drought stress | absent | 16695958 ab | 2068492 ab | 406432 cd | 803315 cd | 19815 bc |
|  |  | presence | 2006583 a | 1748694 abc | 1046232 b | 271558 d | 28862 a |
| *T. serpyllum* | Regular watering | absent | 2232737 d | 411030 def | 249766 cde | 2730913 a | 5131 f |
|  |  | presence | 9406598 c | 325852 ef | 349854 cd | 2213530 ab | 10965 def |
|  | Drought stress | absent | 5807937 cd | 2243451 a | 221050 cde | 2261896 ab | 12217 c-f |
|  |  | presence | 16948574 ab | 2271076 a | 1536494 a | 1405150 bc | 19289 cd |
| *T. kotschyanus* | Regular watering | absent | 1402722 d | 44882 f | 1455 e | 2303626 ab | 8776 ef |
|  |  | presence | 4114605 d | 686712 def | 4669 e | 2211333 ab | 6111 f |
|  | Drought stress | absent | 1234383 d | 1058502 cde | 5062 e | 1026372 cd | 12170 c-f |
|  |  | presence | 15734004 ab | 1284189 bcd | 473465 c | 646397 cd | 28655 ab |

**Supplementary Table S5C.** Phenolics content (peak area mg FW^-1^) in thyme species after drought and herbivory treatments.

| Specie | Irrigation regimes (%fc) | Herbivory | Apigenin-glucuronide | Luteolin-glucuronide | 555-359 | 307-263 | 399-161 |
| --- | --- | --- | --- | --- | --- | --- | --- |
| *T. vulgaris* | Regular watering | absent | 3165627 a | 2905290 bc | 2800684 bc | 16249 a | 339609 b |
|  |  | presence | 3342535 a | 2374402 c | 2524895 c | 13435 ab | 332408 b |
|  | Drought stress | absent | 3934079 a | 3829024 a | 3671104 ab | 14875 a | 270651 bc |
|  |  | presence | 4057360 a | 3377464 ab | 4224009 a | 8341 bcd | 1354477 a |
| *T. serpyllum* | Regular watering | absent | 617555 b | 659887 f | 26028 d | 7647 bcd | 53232 de |
|  |  | presence | 526975 b | 515893 f | 350831 d | 5076 d | 84326 de |
|  | Drought stress | absent | 558873 b | 1046369 def | 346413 d | 7169 cd | 19775 de |
|  |  | presence | 746636 b | 1487837 de | 680711 d | 6411 cd | 137060 bcd |
| *T. kotschyanus* | Regular watering | absent | 1134689 b | 958908 def | 8397 d | 8049 bcd | 9274 de |
|  |  | presence | 600451 b | 778629 ef | 9378 d | 11667 abc | 10438 de |
|  | Drought stress | absent | 468536 b | 400811 f | 5257 d | 8680 bcd | 1590 e |
|  |  | presence | 844661 b | 1595271 d | 871716 d | 7299 cd | 175841 bcd |

**Supplementary Table S6**. Concentrations of phytohormones (ng g FW^-1^) in thyme species after drought and herbivory treatments.

| Specie | Irrigation regimes (%fc) | Herbivory | SA | SA-Gluc | ABA | Cis-OPDA | JA | JA-Ile | OH-JA-Ile | OH-JA | COOH-JA-Ile | Sulfo-JA |
| --- | --- | --- | --- | --- | --- | --- | --- | --- | --- | --- | --- | --- |
| *T. vulgaris* | Regular watering | absent | 77 c | 29121 d | 45 d | 854 d | 532 e | 3.20 d | 2.35 c | 147033 bcd | 1.36 e | 10249442 c |
|  |  | presence | 3807 b | 348471 cd | 955 b | 41593 a | 26091 a | 5900.78 a | 1724.41 a | 428926 a | 187.99 a | 28055307 a |
|  | Drought stress | absent | 51 c | 235169 cd | 46 d | 1653 d | 1156 e | 5.18 d | 3.05 c | 180998 bc | 3.67 e | 14739055 b |
|  |  | presence | 5784 ab | 679581 b | 869 b | 38732 ab | 19307 bc | 4513.58 ab | 1020.29 b | 367460 a | 135.10 b | 28594930 a |
| *T. serpyllum* | Regular watering | absent | 162 c | 108668 cd | 144 cd | 1921 d | 129 e | 3.38 d | 2.66 c | 31580 d | 1.20 e | 5614 e |
|  |  | presence | 3282 b | 305365 cd | 958 b | 29209 bc | 11399 d | 2230.75 c | 302.32 c | 188476 b | 25.40 de | 14602 e |
|  | Drought stress | absent | 220 c | 269720 cd | 216 cd | 1827 d | 241 e | 2.59 d | 5.40 c | 39491 cd | 0.65 e | 6082 e |
|  |  | presence | 6426 a | 1278836 a | 1994 a | 31287 abc | 22555 ab | 4706.48 ab | 262.73 c | 495371 a | 45.77 e | 12140 e |
| *T. kotschyanus* | Regular watering | absent | 270 c | 107507 cd | 196 cd | 1233 d | 595 e | 11.34 d | 3.78 c | 62162 bcd | 1.92 e | 1352185 e |
|  |  | presence | 3674 b | 421472 bc | 542 bc | 26103 c | 11308 d | 3297.96 bc | 1765.83 a | 145894 bcd | 84.29 cd | 3261997 de |
|  | Drought stress | absent | 233 c | 82720 d | 238 cd | 947 d | 212 e | 5.80 d | 5.37 c | 62671 bcd | 2.15 e | 939462 e |
|  |  | presence | 6607 a | 1084063 a | 1929 a | 28032 bc | 15170 cd | 3875.99 b | 345.03 c | 491090 a | 39.80 de | 5799793 d |

**Supplementary Table S7.** The results of the principal components analysis for phenolic compounds of the thyme species.

| PC3 | PC2 | PC1 | Principal component |
| --- | --- | --- | --- |
| 2.08 | 3.33 | 7.97 | Eigenvalue |
| 13.05 | 20.84 | 49.82 | Percent of Variance |
| 83.72 | 70.67 | 49.82 | Cumulative Percentage |
|  | | | |
| PC3 | PC2 | PC1 | Principal component for phenolics |
| -0.32516 | -0.198879 | 0.203638 | Coumric acid |
| -0.344889 | -0.0658868 | 0.252581 | Caffeic acid |
| -0.211385 | -0.431415 | 0.173445 | Ferulic acid |
| 0.370671 | 0.110583 | 0.264237 | Chlorogenic acid (5-CQA) |
| 0.428063 | -0.375891 | 0.106627 | Chlorogenic acid (4-CQA) |
| 0.434846 | -0.361774 | 0.120686 | Chlorogenic acid (3-CQA) |
| -0.053146 | -0.0543572 | 0.343591 | Rosmarinic acid |
| -0.121332 | 0.0345144 | 0.247049 | Quinic acid |
| -0.297848 | -0.247985 | 0.217061 | Rosmarinic acid glucoside |
| -0.177856 | 0.00988039 | -0.301563 | Citric acid |
| 0.193567 | -0.176265 | 0.301338 | Apigenin 6,8-di-c-glucoside |
| -0.0153414 | 0.313001 | 0.279889 | Apigenin-glucuronide |
| 0.06025 | 0.237457 | 0.312384 | Luteolin-glucuronide |
| -0.00836996 | 0.2401 | 0.312502 | 555-359 |
| 0.151757 | 0.415663 | 0.12723 | 307-263 |
| -0.136334 | 0.0632016 | 0.266797 | 399-161 |

**Supplementary Table S8.** The results of the principal components analysis for phytohormones of the thyme species.

| PC2 | PC1 | Principal component |
| --- | --- | --- |
| 1.88 | 7.30 | Eigenvalue |
| 18.83 | 73.07 | Percent of Variance |
| 91.90 | 73.07 | Cumulative Percentage |
|  |  |  |
| PC2 | PC1 | Principal component for phytohormones |
| -0.212624 | 0.346081 | SA |
| -0.426498 | 0.285618 | SA-Gluc |
| -0.402994 | 0.304799 | ABA |
| 0.066378 | 0.357007 | Cis-OPDA |
| 0.025112 | 0.364164 | JA |
| 0.0465539 | 0.367136 | JA-Ile |
| 0.415763 | 0.256311 | OH-JA-Ile |
| -0.162984 | 0.339329 | OH-JA |
| 0.40082 | 0.30527 | COOH-JA-Ile |
| 0.493156 | 0.19199 | Sulfo-JA |


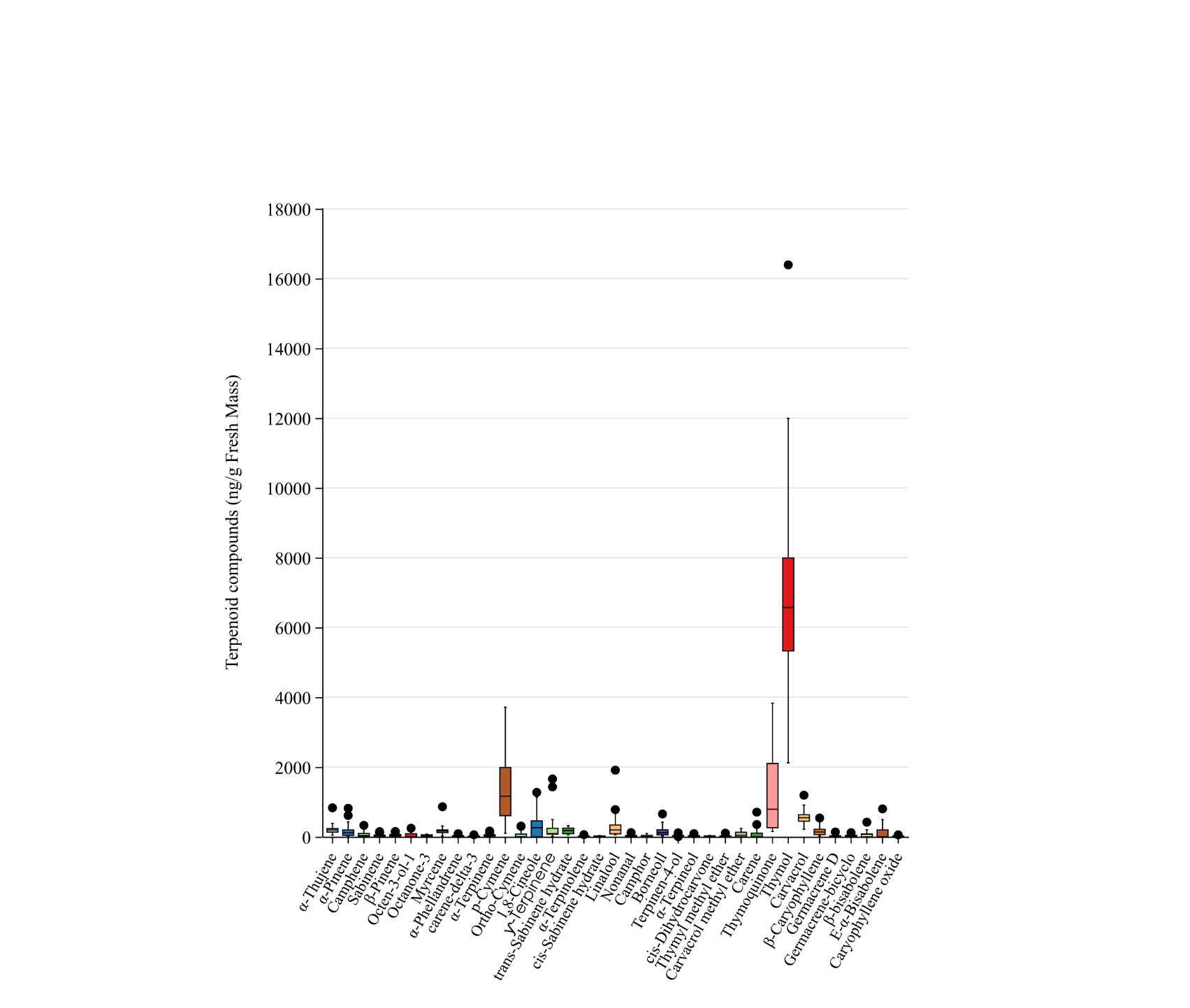
**Supplementary Figure S1.** Variation analysis of terpenoid components from three thyme species (*T. vulgaris*, *T. serpyllum* and *T. kotschyanus*). Terpenoids were measured by GC-MS (The values are expressed in ng g^-1^ of sample fresh mass) and annotated with NIST and ADAMS databases.


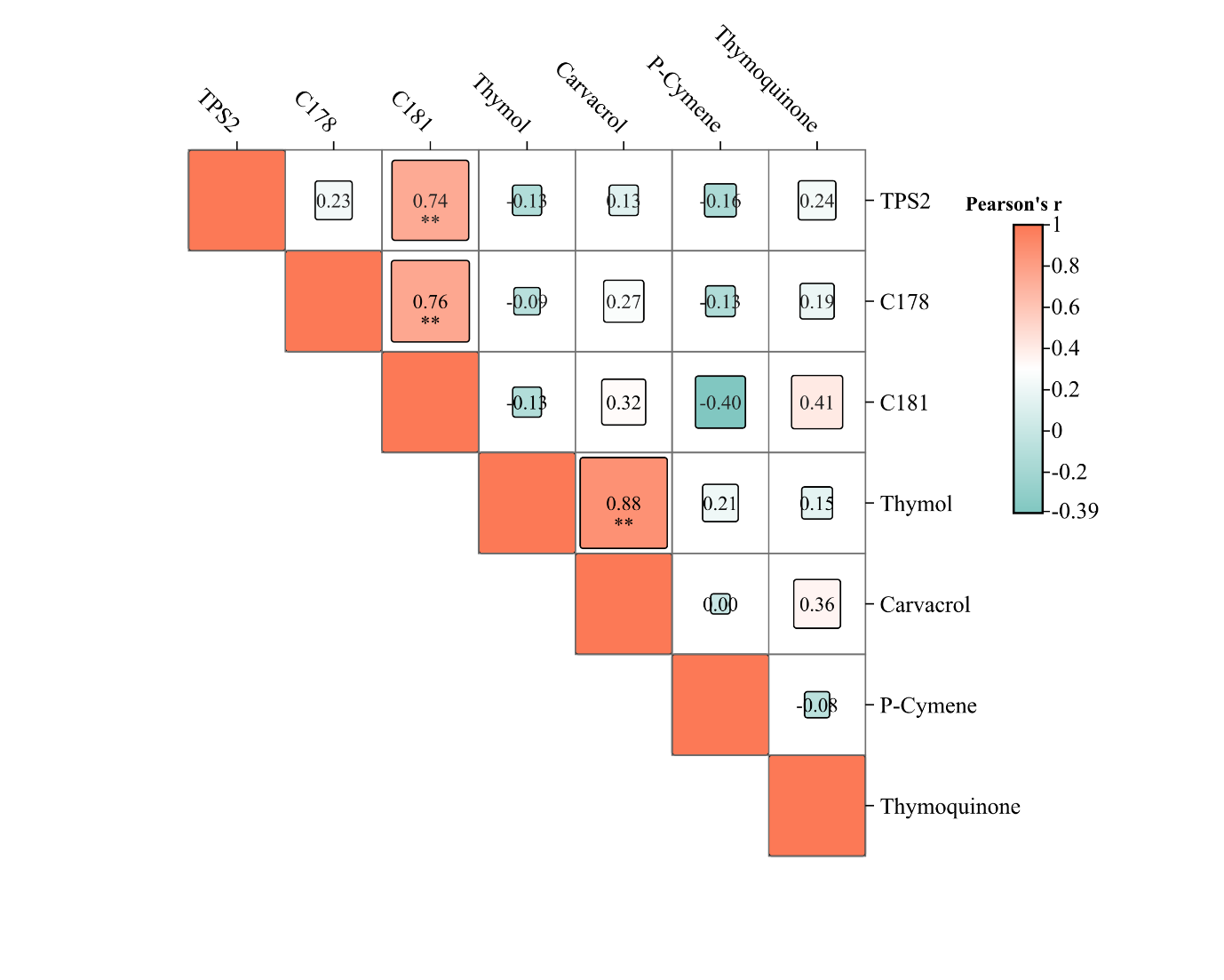


**Supplementary Figure S2.** Gene expression of key biosynthetic genes of terpenoid biosynthesis and the amount of specific terpenoids do not strongly correlate. Pearson’s correlation coefficients between the main terpenoid components (thymol, carvacrol, *p*-cymene, thymoquinone) and gene expression (*TPS2*, *CYP71D178*, and *CYP71D181*) on studied *Thymus* species (*T. vulgaris*, *T. serpyllum* and *T. kotschyanus*) affected by different irrigation regimes and herbivory. * Correlation is significant at the *p* ≤ 0.05 level. ** Correlation is significant at the *p* ≤ 0.01 level.
